# A new approach to simplify surgical colpotomy in laparoscopic hysterectomy

**DOI:** 10.1007/s10397-015-0929-x

**Published:** 2016-01-12

**Authors:** L. van den Haak, J. P. T. Rhemrev, M. D. Blikkendaal, A. C. M. Luteijn, J. J. van den Dobbelsteen, S. R. C. Driessen, F. W. Jansen

**Affiliations:** Department of Gynecology, Leiden University Medical Center, PO Box 9600, 2300 RC Leiden, The Netherlands; Department of Obstetrics and Gynecology, Bronovo Hospital The Hague, The Hague, The Netherlands; Department BioMechanical Engineering, Delft University of Technology, 2628 CD Delft, The Netherlands

**Keywords:** Laparoscopic hysterectomy, Colpotomy, New technology, Innovation of surgical technique

## Abstract

New surgical techniques and technology have simplified laparoscopic hysterectomy and have enhanced the safety of this procedure. However, the surgical colpotomy step has not been addressed. This study evaluates the surgical colpotomy step in laparoscopic hysterectomy with respect to difficulty and duration. Furthermore, it proposes an alternative route that may simplify this step in laparoscopic hysterectomy. A structured interview, a prospective cohort study, and a problem analysis were performed regarding experienced difficulty and duration of surgical colpotomy in laparoscopic hysterectomy. Sixteen experts in minimally invasive gynecologic surgery from 12 hospitals participated in the structured interview using a 5-point Likert scale. The colpotomy in LH received the highest scores for complexity (2.8 ± 1.2), compared to AH and VH. Colpotomy in LH was estimated as more difficult than in AH (2.8 vs 1.4, *p* < .001). In the cohort study, 107 patients undergoing LH were included. Sixteen percent of the total procedure time was spent on colpotomy (SD 7.8 %). BMI was positively correlated with colpotomy time, even after correcting for longer operation time. No relation was found between colpotomy time and blood loss or uterine weight. The surgical colpotomy step in laparoscopic hysterectomy should be simplified as this study demonstrates that it is time consuming and is considered to be more difficult than in other hysterectomy procedures. A vaginal approach to the colpotomy is proposed to achieve this simplification.

## Introduction

New surgical techniques and technical equipment have attempted to facilitate laparoscopic hysterectomy (LH), after shortcomings of LH in comparison with vaginal hysterectomy (VH) and abdominal hysterectomy (AH) were demonstrated [1]. New alternatives for conventional suturing, such as bipolar coagulation, have improved hemostasis of the uterine and ovarian pedicles [1]. Furthermore, in a systematic review, the superiority of vessel-sealing devices with respect to blood loss and shorter operation time in some abdominal procedures was demonstrated compared to other electrothermical devices [2]. Finally, barbed sutures have been introduced for vaginal vault closing, and this technique appears to be equal compared to standard sutures with respect to time to cuff closing, cuff healing, and sexual function [3]. Although some of these effects are debatable, for instance due to possible contributing factors such as learning curve, they do demonstrate the efforts to facilitate the LH. Certainly, notwithstanding the well-known benefits of LH, VH remains the gold standard for the hysterectomy procedure [1, 4], even though in contrast with this statement, recent studies have shown that LH was associated with shorter hospital stay, less blood loss, and less postoperative pain compared to VH [5, 6]. Yet, LH is still associated with a longer operating time [4, 7]. Furthermore, previous studies have demonstrated that LH is regarded as more difficult when compared to AH and VH [8]. Learning curve issues and implementation errors have contributed to these results. However, there still are technical opportunities to simplify the LH procedure. Our hypothesis is that the colpotomy should be addressed in this context. Colpotomy is part of the final surgical steps in the LH procedure, following the ligation of the uterine arteries, the skeletonizing of the cervix, and the dissection of the bladder from the cervix. These steps are relatively hazardous and time consuming in the procedure. It is in this anatomical area where most of the bleeding and ureter injuries occur [9, 10]. Moreover, the delicacy of laparoscopic surgery in this anatomical area was demonstrated by the initial higher incidence of ureter injuries during LH, which only decreased after a certain learning curve was passed [11]. In this light, an alternative route for colpotomy has been investigated: analysis of the current colpotomy procedure demonstrated that the main difficulties of this surgical step are the limited visibility during colpotomy (due to the anterior view of the endoscope combined with the location of the cervix deep in the pelvis), and the need for a 360° circular cutting motion during colpotomy. To overcome these difficulties, a vaginal approach to the colpotomy was suggested. The first test with a prototype of a vaginal colpotomy device on an in vitro vaginal model demonstrated a significant reduction of colpotomy time [12].

The aims of this study were to substantiate our hypothesis and to further evaluate the possibilities of a vaginal approach to colpotomy. The experienced difficulty, the duration of the surgical colpotomy step, and possible agents of change are evaluated. In addition, the idea of a vaginal approach to colpotomy is shaped into a new surgical instrument that may simplify colpotomy [13].

## Materials and methods

**Fig. 1:**
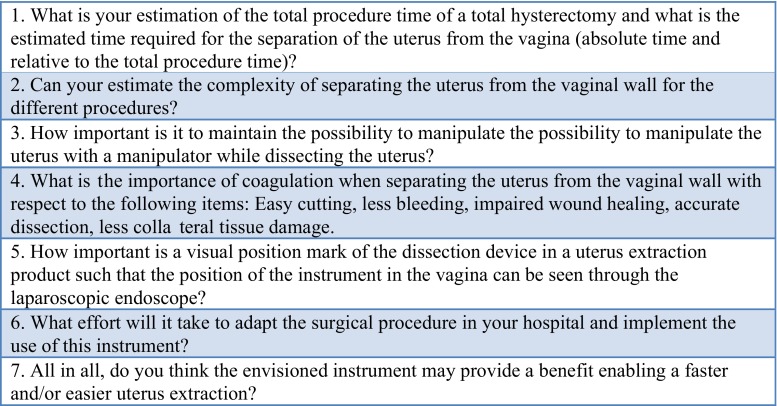
Structured interview

Firstly, to investigate the difficulty of the colpotomy procedure, a structured interview was performed among experts in minimally invasive gynecologic surgery working at different hospitals throughout the Netherlands. The interview assessed the participants perception regarding the surgical step of the colpotomy. Furthermore, they were asked about their opinion regarding several features of the proposed facilitation of the colpotomy. (Figure 1) Participants were asked to answer using a 5-point Likert scale: 1 meaning “easy”/“not important”, to 5 meaning “complex”/“important.”

Next, a prospective cohort study was performed at two hospitals specialized in minimally invasive gynecologic surgery. From June 2010 to May 2014, LH procedures were timed to assess the duration of colpotomy. The total operating time (TOT) was defined as the time from the insertion of the Veress needle to the final stitches used for closing last trocar incision site. Colpotomy time (CT) was defined as the time from the first incision in the vaginal fornix (after ligating the uterine arteries and all uterine ligaments) until the complete separation of the cervix from the vaginal wall. An extrafascial technique was used to perform total laparoscopic hysterectomy. The vaginal wall was opened anteriorly at the vesicovaginal fold, after which the colpotomy was completed. All consecutive LH procedures were eligible for inclusion. This study was exempt from approval by the medical ethics committee. Procedures were performed by five gynecologists who perform LH on a regular basis and have experience in well over 100 TLH procedures. The number of participating gynecologists was chosen to enhance the external validity of the outcome. Inter-surgeon variability was minimized by using similar surgical procedure protocols. Furthermore, all surgeons received their training at the Leiden Residency Program. The Valtchev or Clermont Ferrand uterine manipulator was used. Bipolar and ultrasonic instruments were used for colpotomy. Basic patient characteristics were gathered. The uterine weight and the total amount of blood loss were measured in the operating room. Patients were excluded in case of missing colpotomy time. Complications were classified according to the severity of the complications on the basis of the framework set by the Dutch Society for Obstetrics and Gynecology (NVOG) [14].

### Statistical analysis

Baseline characteristics were summarized by means and standard deviations and, when applicable, by numbers and percentages. For the structured interview, an independent sample *t* test and a paired *t* test were used to compare experts versus residents and the type of hysterectomy, respectively. For the prospective study, *t* tests were used when applicable. A Pearson’s correlation coefficient and analysis of variance (ANOVA) techniques were used to test any correlation between different variables and colpotomies. A generalized linear model was performed to assess the independent effect of certain parameters (such as uterine weight, body mass index (BMI)) on the duration of colpotomy. All tests were performed at the .05 level of significance. SPSS 20 was used to analyze all data.

## Results

### Structured interview

Sixteen experts from 12 hospitals were interviewed (Tables 1 and 2). On average, the experts performed 35 (SD 24) hysterectomy procedures annually, of which 59 % (SD 24) LH procedures, 19 % (SD 21) VH, and 22 % (SD 15) AH. The estimated TOT is 114 (SD 24) minutes, and they estimated this to spend 18 % (SD 11) on the colpotomy. The colpotomy in LH received the highest scores for difficulty (2.8 ± 1.2), compared to AH and VH. Colpotomy in LH was estimated as more difficult than in AH (2.8 vs 1.4, *p* < .001). The same trend is seen for the difficulty of colpotomy in LH versus VH (2.8 vs 2.0); however, this difference was not significant (*p* = .08). With respect to the vaginal approach to simplify colpotomy, the following functions of the envisaged instrument were regarded as moderately important to important by the participants: the ability to manipulate the uterus (4.5, SD 1.4), the presence of coagulation to stop bleeding during the colpotomy procedure (4.2, SD 1.1), and the existence of markings on the device to help visualize the device by the camera (4.6, SD .7).

**Table 1:** Participants opinion regarding colpotomy (*N* = 16 expert)

	Mean (SD)	*p* value
Number of hysterectomy procedures per year	35 (24)	
Amount of TLH (%)	59 (24)	
Amount of VH (%)	19 (21)	
Amount of AH (%)	22 (15)	
Estimated length of TLH procedure (minutes)	114 (24)	
Estimated colpotomy time TLH (minutes)	20 (10)	
Complexity of colpotomy TLH^a^	2.8 (1.2)	
Complexity of colpotomy VH^a^	2.0 (1.3)	
Complexity of colpotomy AH^a^	1,4 (.6)	
Estimated colpotomy vs total OR time (%)	18 (11)	
TLH vs VH	2.8 vs 2.0	.08
TLH vs AH	2.8 vs 1.4	< .001
VH vs AH	2.0 vs 1.4	.02

*TLH* total laparoscopic hysterectomy, *VH* vaginal hysterectomy, *AH* abdominal hysterectomy

Vaginal hysterectomy

^a^1 easy–5 complex

**Table 2:** Preferred functions and adaptation of the new device (*N* = 16)

	Mean	SD
Importance of a uterine manipulator	4.5	1.4
The Importance of coagulation instead of cutting when separating the uterus from the vagina -Collateral tissue damage -Easy cutting -Wound healing -Accurate dissection -Bleeding		
	2.3	1.6
	3.5	2.0
	2.6	1.6
	3.1	2.2
	4.2	1.1
Importance of markings so that a vaginal instrument is visible during laparoscopy	4.6	.7

Scale 1–5 = not–moderate–important

### Colpotomy analysis

Out of 164 consecutive patients, 107 patients undergoing LH were included. Fifty-seven (35 %) were excluded due to missing colpotomy time. Patient characteristics and procedure data are shown in Table 3. Most common indications for surgery were abnormal bleeding and/or uterine myoma. The mean total operating time was 116.4 min (SD 35.3 min), and the mean colpotomy time was 17.9 min (SD 7.8 min). On average, 16 % of the total procedure time was spent on colpotomy. BMI was positively correlated with colpotomy time (.320 and .311, both *p* = .001), and the generalized linear model confirmed the identified correlation and proved that it was independent from the other variables (Table 4). No statistically significant correlation was found between colpotomy time and uterine weight or blood loss.

**Table 3:** Patient characteristics and procedure data (*N* = 107; 91 Leiden University Medical Center and 16 Bronovo hospital)

		Mean	SD	*p* value
Age (years)		49.4	10.6	
BMI (kg/m^2^)		27.4	7.0	
Parity^a^		2	1.4	
		Number (%)		
Previous operations	None	66 (62)		
	One or more abdominal surgeries	41 (38)		
Indication for operation	Abnormal bleeding and / or uterine leiomyoma	68 (64)		
	(pre-)malignancy	37 (35)		
	Other^b^	2 (2)		
Total operating time (min)		116.4	35.3	
Colpotomy time (min)		17.9	7.8	
TOT minus CT (min)		98.5	31.5	
Uterine weight (g)		242.8	175.0	
Estimated blood loss (ml)		142.5	194.7	
Complications (total and %)	Peri-operative lesions^c^	1 (1 %)		
	Post-operative infection^d^	6 (6 %)		
	Other^e^	9 (9 %)		
Colpotomy-total OR time (%)		16	5	
Colpotomy time	No complications occurred (*N* = 91)	18.0	8.1	
	A complication occurred (*N* = 15)	17.9	6.0	1.0
Colpotomy time	No previous abdominal surgery	17.6	7.3	.6
	With previous abdominal surgery	18.4	8.6	

*BMI* body mass index

^a^Median

^b^1 endometritis and salpingitis, 1 abdominal pain

^c^1 bladder injury

^d^5 urinary tract infections, 1 pneumonia

^e^1 ileus, 1 urinary retention, 1 re-admittance for unexplained fever, 1 lost needle during surgery resulting in enlargement of the trocar incision, 1 patient with facial subcutaneous emphysema that required admittance at the intensive care unit, 1 infected hematoma, 1 vaginal cuff dehiscence occurring 4 weeks after surgery, 1 abdominal pain that led to additional surgery 10 days after TLH resulting in a partial oophorectomy, and 1 repeat laparoscopy on the same day regarding a loss of blood exceeding 300 ml

**Table 4:** Pearson correlation and generalized linear model (*N* = 107; 91 LUMC and 16 Bronovo)

	Colpotomy time (min)		
	Pearson correlation	Sig.	*N*
BMI (kg/m^2^)	.329	.001	104
Age (years)	.278	.004	107
TOT minus CT (min)	.380	.000	105
Uterine weight (g)	.092	.349	105
Estimated blood loss (ml)	.082	.399	107
	Generalized linear model B^a^		
BMI (kg/m^2^)	.403	<.001	
Uterine weight (g)	−.002	.703	

*BMI* body mass index

^a^B unstandardized regression coefficient

## Discussion

This study demonstrates that the surgical colpotomy is a time-consuming step in the LH procedure, that is preceded by the hazardous dissection of the uterine arteries, bladder, and cervix, risking blood loss and ureter injuries. Colpotomy time comprises 16 % of the total operation time, even reaching 45 %. Albeit an extreme value, it does demonstrate the difficulty that can be experienced when performing this task. This is substantiated by our structured interview. In accordance with a previous study [8], our structured interview revealed that experts find colpotomy in LH significantly more difficult than in AH, and that the same trend is seen for colpotomy in LH compared to VH (although not significant). It is also demonstrated that a rise in BMI proved to be associated with a longer colpotomy time. This effect of BMI on the duration of surgery is in line with other studies [15, 16]. However, in our study, the effect of BMI on the colpotomy time remained even after correcting for total operation time. Apparently, higher BMI apart from the additional procedure time, accounts for an additional complicating factor regarding the colpotomy step. These women especially may benefit from the simplification of this procedure. Moreover, as the incidence of obesity is increasing, higher BMI will become part of everyday work in laparoscopic surgery [17]. No other factors, such as the amount of blood loss, previous abdominal surgery, or the presence of complications seemed to influence the duration of colpotomy. Surprisingly, also for uterine weight no correlation was found with colpotomy time. It is our opinion, that the colpotomy procedure can be regarded as independent from “uterine” factors, such as uterine weight. Indeed, when performing the colpotomy after all uterine ligaments and arteries have been dissected, the obtained additional mobility of the uterus will compensate for restrictions due to uterine weight. However, although uteri weighing up to 930 g were removed, the vast majority of uteri in our cohort weighed below 360 g. Therefore, we realize that, based on the results from our cohort, our statement may not fully apply to very large uteri. Yet, support of our opinion can be found in literature, where the feasibility of LH in women with larger uteri has already been established [18, 19]. A limitation of our study is the high number of exclusions, especially given the prospective design of this study. However, the overall effect of the exclusions on the outcome of our study is limited. Missing data can be considered random and therefore effect cohort size rather than the results, although the introduction of bias cannot be fully excluded. Only one surgical protocol was used for our prospective study, and this raises the question of external validity regarding other surgical protocols. However the relative colpotomy time that resulted from our prospective study matches the estimated relative colpotomy time from our interview (16 % vs 18 %, respectively), in which gynecologists participated who use different protocols. This study did not focus on procedural steps of the LH other than colpotomy, which could be considered a flaw. For instance, dissection and sealing of the uterine artery would have been an interesting addition. On the other hand, this step has already been enhanced by new surgical techniques and technology. All other steps of the hysterectomy procedure are relatively straightforward and appear to be in no apparent need of improvement. Notwithstanding these shortcomings, our findings regarding colpotomy time are important. A recent study demonstrated that operative time was an independent predictor of postoperative morbidity and reoperation [20]. Furthermore, a cost analysis of different approaches to hysterectomy showed that patient operation room costs and total patient costs are higher for LH when compared to VH, and that longer operation time proved to be an important contributor to these higher costs [21]. In light of these studies, reducing CT and thereby the TOT may have beneficial effects on patient morbidity as well as on health care costs. This will become increasingly important, since there is an increase of laparoscopic hysterectomy procedures at the expense of the number of vaginal hysterectomies [22].

### Vaginal approach for colpotomy

**Fig. 2:**
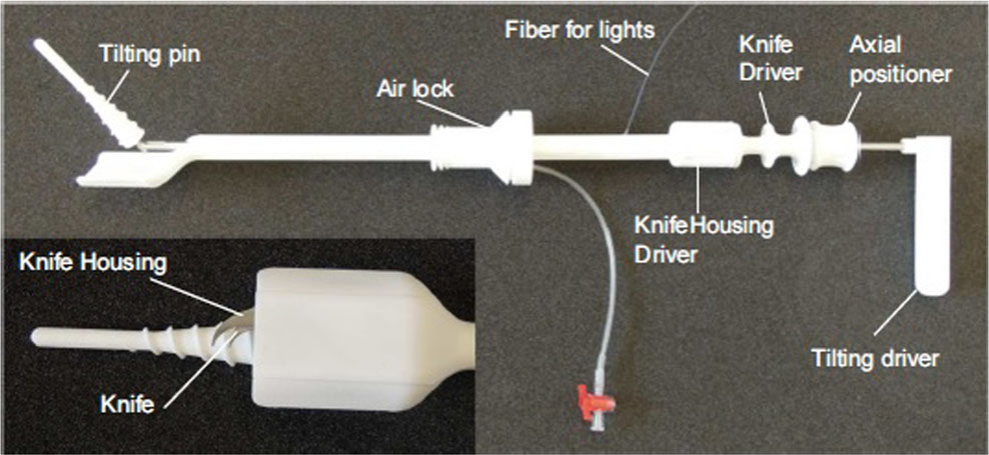
MobiSep prototype

**Fig. 3:**
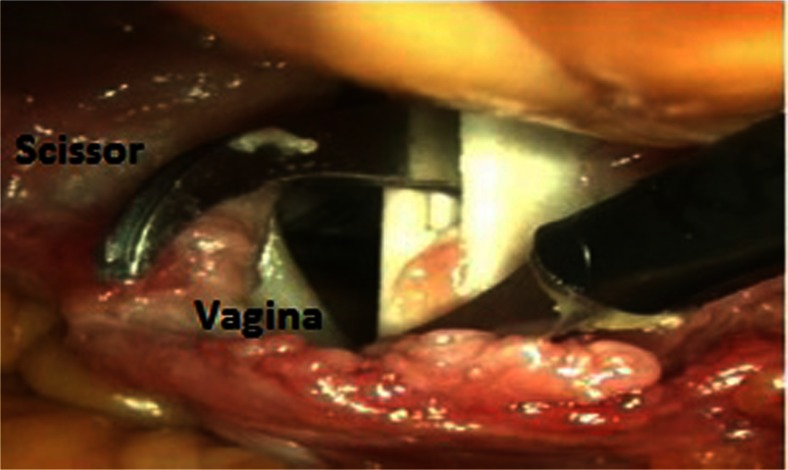
Vaginal colpotomy with MobiSep prototype in human cadaver test

**Fig. 4:**
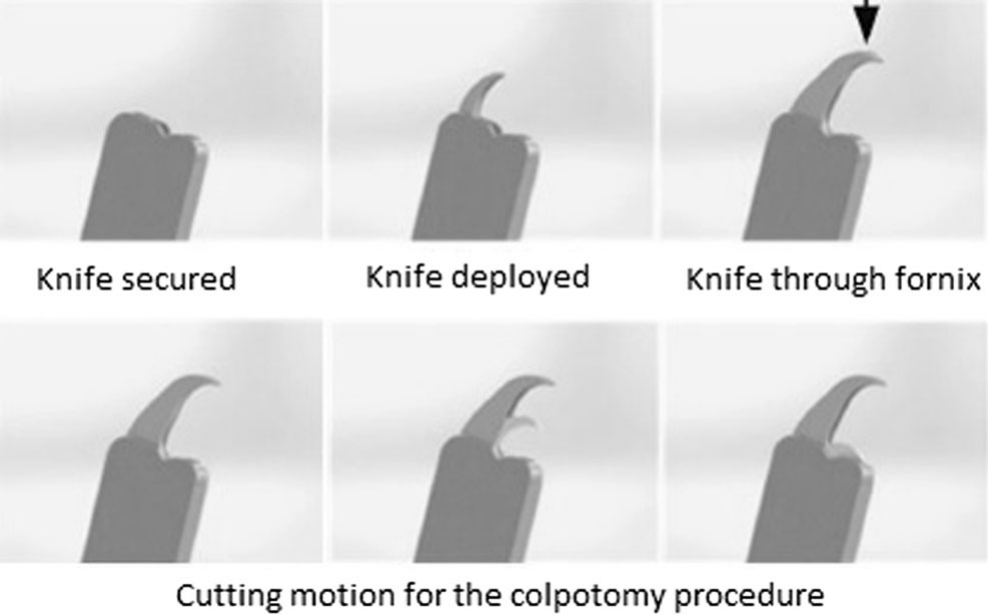
Detail of the knife action of the vaginal colpotomizer in relation to safety cap

A prototype for a vaginal colptomizer device has been assembled [13]. Although several methods exist to perform the surgical colpotomy such as bipolar and harmonics, to our knowledge, the vaginal route to colpotomy has not yet been proposed. Figure 2 demonstrates our prototype. The intrauterine part of the manipulator has mobility in all planes (i.e., anterior-posterior, lateral, and rotation). After introducing the manipulator into the uterus, a cap is positioned over the cervix. This cervical cap, which rotates, has several functions: it presents the vaginal cuff and helps to push the uterus cranially. Furthermore, it houses the knife that enables the vaginal colpotomy. The knife is deployed and operated by moving the knife driver and the handle of the manipulator. The exact location where the knife is introduced into the vaginal wall (and hence in the abdominal cavity) is identified by a light source in the manipulator. Figures 3 and 4 demonstrate the knife during colpotomy in a human cadaver test and in detail, respectively. Finally, after colpotomy is completed, the entire surgical specimen and the manipulator are removed. Certain questions remain to be answered. For instance, our interview tried to assess the preference for a coagulation-based or “cold knife”-based cutting mechanism. Coagulation was preferred in case of bleeding and, to lesser extent, to facilitate the cutting action. However, some concerns were raised over the possible negative effects of coagulation with respect to wound healing. Several studies have reported a higher incidence of vaginal vault dehiscence after LH when compared to VH and AH [23–25]. It has been suggested that electrocoagulation may be the cause for this higher incidence, due to more extensive tissue damage and/or suboptimal tissue healing [26, 27]. However, in large series, no effect of electrocoagulation was demonstrated with respect to the occurrence of vaginal vault dehiscence [28]. Moreover, no effect of the power settings was observed [28]. It was concluded that the current available scientific evidence does not support one technique over the other, and it is expected that this topic will continue to be a main point of interest for gynecological societies. However, in light of the feasibility of the device, a cold knife cutting mechanism was designed. The structured interview also demonstrated the need for a manipulator function integrated in the device. The importance of a uterine manipulator during LH has been demonstrated in literature. A manipulator is considered to increase the distance between the ureter and uterine arteries, thereby creating more space for the dissection of the uterine arteries [29]. Furthermore, in a recent Delphi study, full agreement was reached regarding the use of a uterine manipulator during LH to prevent ureter injuries during LH [30]. This resulted in the final design of the prototype: a uterine manipulator with an integrated vaginal colpotomizer.

In all, the significance of the present study is the clinically driven approach to the innovating the difficult surgical colpotomy step. Experiences in the past have shown the need for a careful introduction of new technology in daily practice [31, 32]. Consequently, innovation should start with a thorough analysis of the problem at hand. The eVALuate study has taught us that LH has certain disadvantages with respect to patient safety when compared to VH and AH [1]. Technical developments have already contributed to the enhanced safety of LH. However, further simplifying the LH is necessary, since our study demonstrates that the surgical colpotomy step takes place in an anatomical area which is at risk for complications, is regarded as difficult, and comprises a considerable amount of the total duration of the LH procedure. Therefore, much can be gained by simplifying this step.

## Conclusions

Earlier studies have taught us that LH has certain disadvantages with respect to patient safety when compared to VH and AH. Technical developments have already contributed to the enhanced safety of LH. However, further simplifying the LH is necessary, since reducing the operation time of LH may reduce health care costs and complication rates [20, 21]. Our study demonstrates that the colpotomy step in LH should be simplified. Not only is this surgical step time consuming bu it is also regarded as significantly more difficult when compared to AH. A vaginal approach of the colpotomy step may solve these issues. A surgical instrument was designed as a uterine manipulator with an integrated vaginal colpotomizer. The device intends to address the shortcomings of the current colpotomy technique. Clinical studies will commence shortly to evaluate the efficacy and safety of the vaginal approach to colpotomy.
